# The impact of organizational culture and leadership climate on organizational attractiveness and innovative behavior: a study of Norwegian hospital employees

**DOI:** 10.1186/s12913-022-08042-x

**Published:** 2022-05-13

**Authors:** Barbara Rebecca Mutonyi, Terje Slåtten, Gudbrand Lien, Manel González-Piñero

**Affiliations:** 1 School of Economics, Innovation and Technology, Kristiania University College, Oslo, Norway; 2 Inland School of Business and Social Science, Inland Norway University of Applied Sciences, Lillehammer, Norway; 3 Department of Economics, Faculty of Economics and Business, University of Barcelona, Barcelona, Spain; 4 Research Centre for Biomedical Engineering, Technical University of Catalonia, Barcelona, Spain

**Keywords:** Organizational culture, Organizational climate, Internal market-oriented culture, Support for autonomy, Organizational attractiveness, Innovative behavior, Hospital employees

## Abstract

**Background:**

In the domain of health services, little research has focused on how organizational culture, specifically internal market-oriented cultures (IMOCs), are associated with organizational climate resources, support for autonomy (SA), and whether and how IMOCs and SA are either individually or in combination related to employee perceptions of the attractiveness of the organization and their level of innovative behavior. These knowledge gaps in previous research motivated this study.

**Methods:**

A conceptual model was tested on a sample (*N* = 1008) of hospital employees. Partial least-squares structural equation modeling (PLS–SEM) was employed to test the conceptual models, using the SmartPLS 3 software. To test the mediator effect, a bootstrapping test was used to determine whether the direct and indirect effects were statistically significant, and when combining two tests, to determine the type of mediator effect.

**Results:**

The results can be summarized as four key findings: i) organizational culture (referring to an IMOC) was positively and directly related to SA (*β* = 0.87) and organizational attractiveness (*β* = 0.45); ii) SA was positively and directly related to both organizational attractiveness (*β* = 0.22) and employee individual innovative behavior (*β* = 0.37); iii) The relationships between an IMOC, SA, and employee innovative behavior were all mediated through organizational attractiveness; and iv) SA mediated the relationship between the IMOC and organizational attractiveness as well as that between the IMOC and employee innovative behavior.

**Conclusions:**

Organizational culture, IMOC, organizational climate resources, and SA were highly correlated and necessary drivers of employee perceptions of organizational attractiveness and their innovative behavior. Managers of hospitals should consider IMOC and SA as two organizational resources that are potentially manageable and controllable. Consequently, managers should actively invest in these resources. Such investments will lead to resource capitalization that will improve both employee perceptions of organizational attractiveness as well as their innovative behavior.

**Supplementary Information:**

The online version contains supplementary material available at 10.1186/s12913-022-08042-x.

## Background

Owing to the fast-paced growth of demands for healthcare and the advancements of health technology, hospital organizations now face increased demands for health services [1], with improvements in areas such as value, efficiency, and innovative thinking [1–3]. Increased patient needs and demand for care are pushing health organizations to keep pace with the increasingly challenging health industry and the complexity of offering quality healthcare [1, 4]. Employee innovativeness is not only becoming essential for addressing patient needs and demands [5], but also indispensable for a continued expansion and adoption of novel ideas in response to current environmental and market changes [6, 7].

With “healthcare’s complex environment and its diverse communities of practice (CoPs)” [5], the role of hospital employees in all categories and their innovative behavior is a key strategic tool in responding to the above challenges [1]. The importance of hospital employees is their knowledge-based patient-oriented expertise [8]. As hospital employees stand at the front line of patient care, their diverse CoPs require an organizational culture and climate that is conducive to employee innovativeness [1, 5, 9] to improve organizational performance, service quality delivery, and patient care [9]. Consequently, given their employees critical role, health organizations should strive to promote a culture and climate that fosters employee innovation at work, here termed “individual innovative behavior (IIB)” [10].

This paper explores whether and how hospitals cultivate an organizational culture and climate that encourages and develops innovative behavior by employees. Organizational culture and climate concern how organizational participants observe, experience, and make sense of their work environment [11]. A positive and healthy interplay between organizational culture and climate may have practical implications for the management of effective organizations. While “climate” refers to experiential descriptions or perceptions of events, culture helps to define why they happen [12–14]. Culture represents an evolved context embedded in systems. It is more stable than climate, has strong roots in history, is collectively held, and is resistant to manipulation [15, 16]. Climate resides within individual perceptions of the organizational context, and when these perceptions are shared across individuals, a higher-level social construct emerges [17, 18]. On the other hand, culture is the property of the collective [19], reflecting deeper phenomena based on symbolic [20] and shared meanings about core values, beliefs, and underlying ideologies and assumptions [15, 21].

Hewko [5] recently argued that while encouraging employee innovation remains an ongoing challenge, “without engaging in critical or creative thinking, hospital employees may find it difficult to identify what, where, and how new ways of working (i.e., innovations) can be introduced.” To this end, this study focuses on concepts that are seldom researched in the domain of healthcare service research, to examine and demonstrate IIB as an indispensable ingredient in improving patient care, hospital efficiency, service quality, service delivery, and other aspects [5, 22, 23].

In this study, organizational culture is reflected in the concept of “internal market-oriented culture” (IMOC), which is purposely or intentionally directed toward employees [24]. Previous research reveals that IMOCs are related to concepts such as employee job satisfaction, turnover intention, work engagement, and organizational attractiveness [10, 24]. However, regarding the newness of the concept, Slåtten et al. noted, “there is a need for additional research into several aspects related to the concept of IMOC,” [10] suggesting that future research should relate IMOCs to climatic conditions such as leadership styles in hospital organizations. This study follows this recommendation and examines how IMOCs are related to leadership support for autonomy (SA) in hospital organizations. SA reflects employee perceptions of the interpersonal climate between themselves and their nearest leader and the autonomy-supportive leadership. An IMOC and SA represent the culture as opposed to the climate of an organization and share the following features: (i) a focus on organizational help and support for employees and (ii) consideration for them as organizational resources, reflecting that both the IMOC and SA are generally under management control and therefore manageable. This latter aspect assumes the potential to capitalize on IMOC and SA to achieve desirable outcomes for the hospital organization.

This study examines two types of capitalization on IMOCs and SA, motivated by previous work by Slåtten et al. [10, 24]. According to them, an IMOC should specifically relate to hospital employees’ perceptions of organizational attractiveness (OA) [10] and employee IIB [24]. In the case of OA, Trybou et al. [25] noted that “hospital attractiveness is of major importance.” Regarding IIB, previous research has found positive innovative attitudes and behaviors to be a vital source of competitive advantage through people [26, 27]. Communities of people evolve, and the roles of the actors within are undergoing change [28]. Collaboration could be formal (work groups or project teams) or informal (such as communities of practitioners or informal networks). Today, research and innovation practices are underway in the practitioner community [29, 30], generating advances and breakthroughs in science, technology, and innovation, opening up opportunities for new interdisciplinary combinations [28, 31, 32]. This level of formality in work groups explains why this study refers to the concept of IIB instead of innovative work behavior (IWB), given that IWB refers to more formal work groups and IIB to less formal ones. Therefore, it is vital that employees can communicate and implement novel ideas through formal and informal means. This study contributes to research on IMOCs and SA in health organizations as antecedents to employee IIB. As Oppi et al. [3] noted, “studies investigating factors that shape innovative behaviour at work are scarce,” especially in health organizations [5, 33].

This paper proceeds as follows. The first part provides the conceptual model of the study. Second, there is a description and definition of each concept, followed by a discussion in which relationships between concepts are hypothesized. Third, a description of the methodology and findings from the empirical study is presented. The paper closes with a discussion of the findings, including several proposals for future research, as well as an overall conclusion of this study.

## Conceptual model of the study

**Fig. 1:**
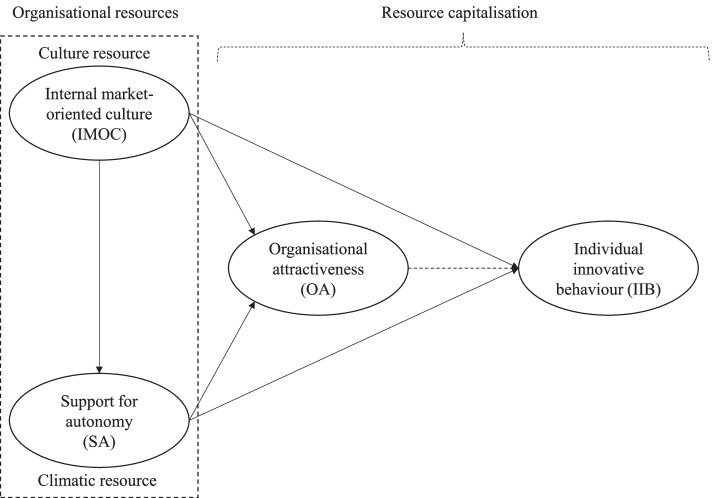
Conceptual model of the study of the relationship between organisational resources and resource capitalisation

The conceptual model of this study is represented in Fig. 1. As seen on the left side of the figure, marked with a dotted line, IMOC and SA represent two distinctive but interrelated types of “organizational resources.” Specifically, an IMOC represents an organizational culture resource, whereas SA is an organizational climate resource. Notably, the term “resource” describes four common characteristics relevant to both IMOCs and SA. First, both constitute relatively intangible organizational resources. Second, neither IMOCs nor SA are static resources but rather are dynamic and subject to change (either positive or negative) as time passes. Third, the term “resource” signals a potential to capitalize on IMOC and SA, which thus have the potential to contribute to competitive advantage of one hospital over others. This latter view is consistent with the resource-based view (RBV) theory [34], which assumes that the constellation of resources is both idiosyncratic and heterogeneously distributed across firms. Fourth, based on the second characteristic, the term “resource” implies the possibility of a hospital organization managing and exerting a degree of control over the two resources (IMOC and SA), proving an opportunity to invest in them. Thus, on this basis and as shown in Fig. 1, the following logic is assumed: If a hospital organization undertakes a “resource investment” in IMOC and SA, the outcome may be capitalization manifested in positive growth in both employees’ perceptions of OA as well as their IIB. Therefore, OA and IIB in Fig. 1 are termed “resource capitalization” because they both stem from and reflect an outcome of the two organizational resources of IMOC and SA.

Above, it is proposed that both IMOCs and SA are directly and indirectly related to two types of resource capitalization: (i) hospital employee perceptions of OA and (ii) hospital employees’ levels of IIB. In the following sections, the concepts and linkages between the concepts in Fig. 1 are elaborated in detail.

### Conceptual framework

As depicted in Fig. 1, the conceptual model of this study proposes two organizational resources, IMOC and SA, that fall under the resource categories of organizational culture and organizational climate. Historically, the construct of climate preceded that of culture [35], climate being widely studied as a result of its demonstrable influence on organizational effectiveness [36–38], as well as its relationship to individual motivation and behavior [39, 40]. However, there has been considerable debate about the similarities and differences between the two terms [13, 16, 41]. A major difference lies in the contention that the climate of an organization consists essentially of shared perceptions, whereas the culture of an organization is made up of shared assumptions [42]. In a similar vein, Moran and Volkwein [43] have suggested that climate consists of attitudes and values alone, whereas culture exists as a collection of basic assumptions, in addition to attitudes and values. Recently, scholars have gone a step further, focusing on how and why the two constructs can be linked to provide a more comprehensive and parsimonious view of the higher-order social structure of an organization [12, 44, 45]. This study has focused on the SA as an organizational climate resource, and an IMOC as an organizational culture resource. In line with Fig. 1, the two resources are further elaborated below.

#### Support for autonomy (SA)

Autonomy in a work context “has been consistently associated with individual innovativeness in the workplace” [5]. Previous health services research has revealed the importance of leadership styles and their importance for encouraging innovation at work [23, 46]. Thus, this study has focused on a specific type of autonomy provided by leaders in a workplace, namely SA.

The concept of SA relates to the interpersonal work context in organizations. As shown in Fig. 1, SA is considered to be a climatic resource within the organization, assumed to be under management control and therefore relatively manageable. Specifically, SA concerns employees’ perceptions of “how things are done here” in the hospital organization. SA embraces whether the interpersonal context is “… autonomy-supportive when managers provide a meaningful rationale for doing the tasks, emphasize choice rather than control, and acknowledge employees’ feelings and perspective” [47]. SA reflects employees’ perceptions of a positive and good leadership style, referring to the relationship between employees and their direct leader and whether they perceive this interpersonal context to be encouraging, motivating, and stimulating.

SA originally related to ideas within the domain of self-determination theory (SDT) [48]. Previous research has emphasized SDT as a highly relevant and appropriate framework for studying aspects of motivations in work contexts [49]. In SDT, there are two types of motivation, labeled (i) autonomous and (ii) controlled motivation. Autonomous motivation is an inner or self-driven type of motivation whereby “the person behaves with a full sense of volition and choice” [47]. By contrast, controlled motivation is diametrically opposed to autonomous motivation. It is a non-self-determined type of motivation imposed from without, meaning “that the person engages in an activity with an experience of pressure and control” [47]. This study limits its focus to autonomous motivation. There are four reasons for this perspective in the study of SA. First, and most fundamentally, under SDT as a guiding theoretical framework, SA is closely associated with the autonomous motivation of a person [47, 50]. Second, in work contexts (as in this study), many employees consider autonomy to be a desirable and a preferable condition that they often actively seek in their work role. In line with this, Amundsen states, “individuals who seek autonomy at work are often searching for inner motivational environments and situations that provide them the opportunity for self-determination, initiative and choice” [51]. Third, autonomous motivation is described as the “highest quality of regulation” [47] and is associated with positive outcomes. Fourth, SA is especially effective when individuals perform complex tasks. “Complex tasks require creativity, deep processing of information, and information integration” [52]. This latter aspect, associated with SA, is especially relevant considering the focus on IIB in this paper.

#### Internal market-oriented cultures (IMOCs)

Previous health services research on organizational culture reveal the multifaceted nature of the concept of organizational culture, which collectively have “been extensively studied in relation to individual innovativeness” [5]. This is because culture is commonly found to be key in supporting, encouraging, and fostering employees’ IIB at work [7, 53]. Nonetheless, scholars in health services research have called for further studies to extend our current understanding of the multifaceted concept of organizational culture and its role in encouraging individual innovation at work [1, 4, 54–57]. For example, in examining 331 Chinese nurses, Jing et al. [56] found that organizational culture, studied as workplace fun, was positively correlated with IIB. As such, this study has focused on a specific type of organizational culture attentive to employees, namely IMOC.

The IMOC concept is viewed as an “organizational culture purposely or intentionally directed toward employees in the organization” [10]. Specifically, in this study, an IMOC is presented in Fig. 1 as an organizational culture resource. An IMOC focuses on employees and whether there exists a culture of supportiveness within the hospital organization. There are five reasons for focusing on a supportive organizational culture, here termed an IMOC. First, culture has previously been viewed as an essential part of any organization [58, 59], and as such, a supportive organizational culture emphasizes values that are internal and employee focused [10, 60, 61]. Second, organizational culture is a key factor in better understanding and sustaining “the observable norm-based behavior that constitutes organizational culture” [24]. Third, organizational culture management in health organizations, especially IMOCs, is perceived as an indispensable part of health system reform [58]. Fourth, organizational culture has been linked to better performance and termed *a* powerful determinant of long-term organizational success [58]. This is owing to its “ability to create a sense of identity and rules” [58], which aids organizations, especially health organizations, to achieve their goals. Fifth, hospital employees’ knowledge about their organizational culture is an effective coping mechanism in this current fast-changing environment, because knowledge can offer insights and solutions into the problems health organizations now face [60].

Previous studies show that health organizations that focus on their organizational culture have derived positive outcomes, such as overall quality improvement and positive employee behaviors and attitudes [60, 62]. In addition, previous studies focusing on IMOCs among frontline employees in healthcare found that they have a positive influence on overall job satisfaction [10] and employee engagement [24]. Therefore, previous research has proposed that organizational culture is vital for health organizations [24, 60]. A supportive organizational culture relates to its tangible and visible characteristics. In this study, the IMOC reflects hospital employees’ experiences, beliefs, and expectations regarding their organizational culture. The IMOC focuses on the relationship between the organization and its employees, and whether employees identify the overall conditions of their organization to be motivating, inspiring, and encouraging.

#### Organizational attractiveness (OA)

In this study, the concept of OA centers on whether people perceive the hospital organization to be a great place to work. According to Trybou et al. [25] “hospital attractiveness [is] … of major importance.” In a similar vein, Yan and Kung [63] describe OA as “the core values … especially for the labor-intensive healthcare industry.” Originally, OA stems from and within the domain of employer branding [64]. However, much of previous research on OA has narrowed its focus to external aspects related to recruitment [65] and how companies communicate to prospective applicants that “our” organization is a desirable place to work. It is important to appear attractive and actively “sell” the hospital organization as an attractive organization to work for to potential new candidates. On the other hand, some would say that an even more fundamentally important criterion for OA is the perception of hospital employees that the hospital organization is genuinely attractive, which is the focus of this study. Studies have found that those (current employees) who perceive their organization to be attractive, which is synonymous with the term “a great place to work,” are four times more willing to devote extra effort to their work role [66]. Therefore, and in contrast to the dominant focus in previous research, the emphasis is on OA from an internal and current employee perspective. In line with previous research taking this current employee perspective, OA is considered to be an attitudinal construct [24]. Specifically, OA relates to current employees’ overall attitude concerning whether their organization is an attractive employer [24]. It is notable that the concept of OA potentially contains or embraces both cognitive and affective aspects of employees’ perceptions when considering the attractiveness of their hospital organization. Accordingly, OA reflects current employees’ overall cumulative attitudes and whether they are “viewing the [hospital] organization as a desirable entity” [67].

#### Individual innovative behavior (IIB)

According to Hult et al. [68] innovation in an organization can be manifested in a diversity of ways and places in the organization such as a “new product or service, a new production process, or a new structure or administrative system.” Institutions are forced to innovate at a faster rate to maintain their competitiveness in the market. However, innovation in this study is limited in its scope and perspective in three ways. First, it focuses on innovation associated with and directed toward employees in the hospital organization. This means it is mainly initiated and driven by employees. Second, manifestation of innovation is related to work roles. Third, innovation is studied at the individual employee level. These three aspects collectively encompass IIB.

IIB refers to the use of novel ideas and solutions by employees to solve problems at work [69]. It comprises problem detection, promotion of new ideas, and the actual application of novel ideas or solutions [70]. Therefore, IIB is closely related to everyday practices and employees’ reflections on how things are done and their capability to propose ideas to improve work performance. Accordingly, IIB embraces or functions as a form of “learning and knowledge creation, integrated into daily work practices” [46]. As such, these innovative practices take place in the practitioners’ community [29, 30], generating advances and breakthroughs in real science, technologies, and daily practice [28, 32]. CoPs are promoted in the healthcare sector as a means of generating and sharing knowledge, improving organizational performance [9], and designing frameworks for systematic evaluations of the CoPs’ effectiveness in improving practice and sustaining improvement [71, 72].

CoPs connect with what we understand to be informal as opposed to formal collaboration (formal work groups or project teams). This level of formality in work groups explains the use of the IIB concept instead of IWB, considering that IWB refers to more formal work groups and IIB to less formal ones. IWB is defined as deliberate introduction and solicitation of ideas (whether between individuals, groups, or organization as a whole), processes, products, or procedures that are relatively new to the unit of adoption, and intended to benefit greatly the individuals, groups, or organization [73]. The number of patents/inventions in a country can be increased by employee IWB [74]. Thus, IIB appears to be a more “specific form of change-oriented activity” [75] that reflects employees’ applications of new and useful ideas in their hospital work role.

### Theoretical relationships between concepts

As shown in Fig. 1, SA is assumed to have a direct impact on IIB. Previous research has shown that SA is associated with different types of positive outcomes, including job performance [52, 76]. For example, in a study by Kanat-Maymon and Reizer [52], the authors found that supervisors’ autonomy-supportive managerial style was, among other factors, positively associated with job performance in a sample of sports analysts [52]. Similarly, in a study undertaken in a healthcare setting, Gillet et al. [76] found that supervisor autonomy support was indirectly related to nurses’ job performance. Gillet et al. [76] defined job performance as nurses’ perception of their team’s quality of work. In this study, job performance refers to employees’ IIB. IIB embraces both (implicitly) a person’s cognition (that is, thinking or creativity) as well as the explicit manifestation of a person’s behavior (actually trying out new ideas in practice).

IIB is demanding and can be characterized as a complex task. Previous research suggests that a necessary foundation for performing such complex tasks is autonomy. Kanat-Maymon and Reizer [52] state, “complex tasks … tend to require a higher degree of … autonomy.” According to SDT, SA is “posited to facilitate the needs for autonomy” [47]. On this basis, it is assumed that when employees experience SA from their direct leader it should raise their IIB. Research has yet to examine this linkage in a healthcare context. However, previous research supports a positive linkage between SA and employees’ creative performance [77]. Furthermore, there is empirical evidence that employee autonomy is associated with characteristics such as innovative behavior [78], creative work involvement [79], creative self-efficacy, and innovative activities [80]. Furthermore, a link between SA and IIB is also supported by the LMX (leader–member–exchange) theory. LMX theory builds on social exchange theory [81, 82]. According to LMX theory, a leader has a unique relationship with each member of the organization [79]. It has been suggested that a high-quality relationship between leaders and members in an organization has several positive outcomes, such as improved job performance. Consequently, based on LMX theory, when employees perceive a high-quality SA relationship with their direct leader, it is reasonable to assume that this constitutes a necessary foundation and functions as a promoting factor for employee IIB. Naturally, there is probably variation in employees’ perceptions of SA, ranging from low to high. However, in this study, it is expected that the more SA a direct leader provides to employees, the more it will increase employee IIB. This relationship is stated formally in this hypothesis:

- Hypothesis 1: SA is positively related to employee IIB.

As proposed in Fig. 1, this study proposes two links that interconnect to form a chain. The first link in the chain is a direct relationship between SA and OA. The study proposes that employees’ perception of leadership style (referring to SA) is associated with their attitude toward the attractiveness of the organization. However, this specific relationship is yet to be explored, and the literature provides empirical evidence and documents the finding that leadership style and performance of the leadership role are strongly associated with the employees’ perception of their organization. For example, previous research in healthcare has revealed a significant link from the performance of management tasks and leadership style to employee attitudes. Their attitudes are reflected in measures such as job satisfaction [83], work engagement [84], turnover intentions [85], and a range of other work- and organization-related factors. In this study, employee-perceived OA is defined as an attitude. Consequently, the “list” of employee attitudes influenced by leadership in organizations should also include OA. Therefore, there are good reasons to assume a direct relationship between SA and OA. This view is formulated as the following hypothesis:

- Hypothesis 2: SA is positively related to OA.

As mentioned above, the first proposed link is the relationship between SA and OA. When this first link (SA–OA) is established, it should lead to the second link, which is between OA and IIB. Therefore, as shown in Fig. 1, this study also suggests that the relationship between SA and IIB is mediated through OA. This relationship represents an additional and complementary “route” through which SA promotes IIB as proposed in the first hypothesis. Accordingly, SA of leaders is “a coherent cluster of supervisory behaviors that collectively create that interpersonal tone of support and understanding” [86]. Consequently, when employees perceive the SA of the leader to be favorable and positive, this should strengthen employees’ attitudes toward the attractiveness of the organization that employs them. This positive attitude of employees regarding SA could be manifested in OA expressions such as: “this is a great place to work” or this is a “great employer.” Similar to the relationship between SA and OA, scant previous research has examined the specific association between OA and IIB as a type of job performance. However, the literature supports the view that OA is linked to job performance. For example, in a study undertaken in healthcare settings, Slåtten et al. [24] found that OA was positively significantly associated with job performance. Job performance in this study was reflected in the quality of service provided to hospital patients and employee work engagement [24]. Moreover, Fortune 100’s *Best Companies to Work For* also suggests a link between OA and job performance, stating, “employees who say they have a great place to work [or what this study labels OA] were four times more likely to say they’re willing to give extra to get the job done” [66]. Social cognitive theory (SCT) also provides theoretical support for the assumption of an association between OA and IIB. SCT suggests that “… beliefs and motivations are formed on valuable judgments” [87]. Based on this SCT assumption, there are good reasons to expect that when employees judge OA more positively, it should strengthen their motivation for IIB. The discussion above suggests that OA functions as a link or a mediator between SA and IIB. Specifically, it means positive perceptions of leader SA should lead to more positive attitudes toward employee OA. Next, when OA increases, this should strengthen employees’ motivation to use their capability to experiment with novel ideas and find creative solutions in their work, thus increasing their IIB. This chain of links can formally be stated in the following hypothesis:

- Hypothesis 3: OA mediates the relationship between SA and employee IIB.

Figure 1 also suggests that an IMOC has a direct impact on SA. Simultaneously, SA is assumed to function as a mediator between IMOC and both OA and IIB. The idea for both of these relationships assumes some fundamental conditions that cultivate and promote SA and in the next round may have an impact on both perceived OA and employee IIB. This “engine” or prerequisite that triggers and initiates this domino effect is suggested to be an IMOC. An IMOC, as mentioned above, is defined and described as a type of organizational culture. Previous research has well documented and emphasized the fundamental importance and role of organizational culture. Organizational culture is said to “pervade all aspects of organizational life” [59]. When focusing on culture in organizations, one looks at “more fundamental characteristics of organization” [88]. The importance of organizational culture lies in its proposition as a “critical first step toward creating a satisfactory work environment” [59]. For example, organizational culture “provides the rules for behavior within organizations” [83]. These “rules,” stemming from organizational culture, are transferred to all organizational members. The transmission is not limited to employees. It also extends to managers and leaders of the organization and serves as a guide to appropriate behavior and work practices. There is a link between organizational culture and leadership behavior. As Banaszak-Holl et al. [59] stated, “organizational culture provides a key mechanism by which top management integrate managerial actions.” Previous research provides good support for the view that there is a positive association between organizational culture and leadership behavior. This positive relationship is also found in healthcare research [83, 89]. Consequently, parallel to findings in previous research, there are good reasons to expect organizational culture in this study to represent the concept of IMOCs, which is positively associated with the SA of leaders. This leads to the following hypothesis:

- Hypothesis 4: The IMOC is positively related to SA.

As mentioned above, an IMOC in an organization provides direction and behavioral codes of conduct for the SA of organization leaders. Consequently, when an IMOC positively increases their SA, this should also increase both employees’ perceived OA and their IIB in an organization. Specifically, in this study, it is expected that the stronger the IMOC of an organization is, the more it drives the SA of leaders and subsequently both the OA and IIB of employees. Accordingly, an IMOC initially functions to promote OA as perceived by employees and their IIB, which work through the mechanism derived from leader SA. Consequently, it is expected that SA has a mediating role between IMOC and both OA and IIB. Based on the above discussion, the following two hypotheses are proposed:

- Hypothesis 5: SA mediates the relationship between an IMOC and OA*.*
- Hypothesis 6: SA mediates the relationship between an IMOC and employee IIB.

According to Fig. 1, IMOC is assumed to have a direct impact on IIB. Previous research has indicated that in health organizations with a supportive organizational culture for employees, it can be a source of competitive advantage [90]. Scott et al. [91] argued that “structural changes alone do not deliver anticipated improvements in quality and performance in health care.” Consequently, organizational culture should be key to improvements in quality and performance for health organizations. For example, Hogan and Coote [92] explored the role of organizational culture in professional service firms and found it to be a key variable in fostering IIB at work. Similarly, Homburg and Pflesser [88] found organizational culture to be directly related to employee performance. In this study, performance refers to employees’ IIB, which encompasses both the production and implementation of novel ideas in a specific work role. Consequently, there are good reasons to assume that an organizational culture that supports new ideas or new ways of accomplishing a work task have the potential to foster employee IIB directly. On this basis, the following hypothesis is suggested:

- Hypothesis 7: An IMOC is positively related to employee IIB.

Furthermore, it is also assumed that the relationship between IMOC and IIB is mediated through OA. As seen in Fig. 1, this relationship represents an indirect and supplementary route of links for IMOCs promoting IIB compared with that proposed in Hypothesis 7. There are two links in this chain. The first link assumes that IMOC has a direct impact on OA. As mentioned above, the concept of OA refers to employees’ attitudes and whether they perceive the hospital organization to be an attractive organization in which to work. It is reasonable to assume that organizational culture, in this study reflected in the IMOC, has a significant impact on employee perceptions of OA. According to Chhabra and Sharma [93], organizational culture is one of the most preferred organizational attributes. Previous research supports the view that employee perceptions of organizational culture are related to employees’ attitudes [4, 60, 94, 95]. A previous study using the concept of IMOC in a healthcare setting has shown that IMOC is directly linked to hospital employee perceptions of OA [24] as well as to employee satisfaction [10]. Consequently, in line with previous research on the impact of organizational culture, and especially those using the IMOC construct, there are good reasons to expect that IMOC is related to OA. This leads to the following hypothesis:

- Hypothesis 8: An IMOC is positively related to OA.

Furthermore, and this leads to the second link between IMOC, OA, and IIB, when employees perceive the organization to be attractive, because of the IMOC, it is also reasonable to assume that this positive attitude (reflected in OA) will motivate and engage employees to devote both more time, more of their mental or physical capacity, and generally more willingness to work to benefit the interests of their hospital organization. This idea and logic are analogous to what was suggested in Hypothesis 3 regarding the impact of SA on OA and IIB. Specifically, based on SCT [87], an IMOC (similar to SA) can boost employee perceptions of OA and strengthen their motivation for IIB. To the authors’ knowledge, no previous research has examined this exact relationship in health services research. However, previous research in hospital settings has identified OA as a mediator between IMOCs and aspects of work role performance such as employee engagement and service quality [24]. Consequently, in line with this research, it is assumed that OA functions as a mediator between IMOC and IIB. This leads to the following final hypothesis:

- Hypothesis 9: OA mediates the relationship between an IMOC and employee IIB.

All the suggested hypotheses guiding this study are summarized in Table 1.

**Table 1:** Hypotheses leading this study

Hypothesized relationships	
H1	SA is positively related to employee IIB.
H2	SA is positively related to OA.
H3	OA mediates the relationship between SA and employee IIB.
H4	The IMOC is positively related to SA.
H5	SA mediates the relationship between an IMOC and OA.
H6	SA mediates the relationship between an IMOC and employee IIB.
H7	An IMOC is positively related to employee IIB.
H8	An IMOC is positively related to OA.
H9	OA mediates the relationship between an IMOC and employee IIB.

*Note*: *SA* Support for Autonomy, *IIB* Individual Innovative Behavior, *OA* Organizational Attractiveness, *IMOC* Internal Market-Oriented Culture

## Methods

### Study design and settings

This study aims to examine the relationship between an IMOC, SA, OA, and IIB, and is part of a larger research project. In February 2020, data were gathered from one of the largest health expert communities in Norway, in the Inland Norway region, extending over 40 sites with over 10,000 employees. With over 10 divisions, the hospitals offer various services in relation to psychiatric and somatic illnesses. Initially, the health expert community in the Inland Norway region was invited to participate in the study. However, following the decisions of the Director of Research (DOR), Human Resources Management (HRM) office, division managers, and department managers, we were granted access to a total of 2000 hospital employees drawn from seven staff units and 10 hospital divisions. Initial contact, and all contact with the hospital, was sought through the DOR. The DOR was given a pitch on the research project, along with its aim, estimated time, and essential resources. The DOR encouraged and motivated the hospital administration to provide access and opportunities for the hospital employees to participate in the research project. In addition, the DOR supported the dissemination of information about the research project and the online questionnaire link. Information about the research project covered its purpose, participants’ rights, time allotted to the questionnaire, a link to the questionnaire and the contact details of the research project leader(s). All contact with the hospital, HRM, division managers, and staff units went through the DOR. Specifically, the DOR forwarded any information to division managers, who forwarded it and the survey to their employees. This was in line with the hospitals’ HRM policy and employee protection policy.

### Study participants

In this study, a hospital employee is understood to be any individual employed by a hospital whose services or labor are provided to a hospital, for which compensation is reflected in the hospital payroll records. As mentioned above, a total of 2000 hospital employees were invited to participate in the study. Out of these and through convenience sampling, a total of 1008 hospital employees participated in the survey, resulting in a response rate of 50.4%. As shown in Table 2, 73% of respondents were female. The high number correlates well with the Norwegian context, where more than 80% of employees of health organizations are women [96]. Moreover, 37.3% were under the age of 45 years, and 77.5% were full-time workers. In addition, over 55% of the participants had amassed substantial work experience because they had been with the organization for more than 10 years. While there were minor differences among divisions, it is important to note that this study focused on individual behavior and not division-level differences. In addition, the study sought to examine all hospital employees regardless of their work roles, so it did not focus on minor differences between staff roles, such as those between nurses and doctors. Therefore, this study offers new insights into issues related to IIB among individual hospital employees.

**Table 2:** Personal characteristics of the study sample (*N* = 1008)

		%
Sex	Female	73.0
	Male	27.0
Staff role:	Nurse	33.0
	Doctor	8.7
	Others (admin. Staff, other health professionals, etc.)	58.3
Employed:	less than 5 years	26.9
	between 6 and 10 years	18.0
	between 11 and 20 years	30.3
	more than 20 years	24.8
Part-time or full-time:	part-time job	22.5
	full-time job	77.5
Age:	younger than 45 years	37.3
	between 46 and 55 years	32.2
	older than 55 years	30.5

### Instruments

The study used four established instruments to measure the conceptual model of the study (Fig. 1): IMOC, SA, OA, and IIB. All items in the survey required participants to respond using a seven-point Likert response scale (1 = strongly disagree to 7 = strongly agree). In addition to the survey statements shown in Table 2, the questionnaire included personal characteristics such as age, sex, type of employment, and work role. To ensure quality in the overall research design, two experts in the field, with 34 randomly selected hospital employees, completed a pre-test. In addition, the survey was conducted in the Norwegian language. For this reason, several workshops with academic experts and employees were held to verify the back-translation. Note that as mentioned above, the survey used in this study is part of a larger survey research project focusing on various aspects of employee relations in health organizations. The statements used in this study are appended (see Additional file 1: Appendix 1).

The IMOC was measured using eight items from Slåtten et al. [24]. SA was measured using five items from Amundsen [51]. OA was measured using two items from Trybou et al. [25]. IIB was measured using five items from Janssen [97] and Scott and Bruce [74]. It is important to note that the items used in this study were adjusted to the context of hospital employees in Inland Norway. All items used in this study are summarized in Table 3.

**Table 3:** Latent variables and items used in the study

Latent variable	Item label	Items
IMOC	IMOC1	Employees have the opportunity to discuss their needs with management.
	IMOC2	Training is seen in the context of individual needs.
	IMOC3	Management spends time talking to their employees when needed.
	IMOC4	Management wants employees to enjoy their work.
	IMOC5	Management shows a sincere interest in any problems faced by employees.
	IMOC6	Management understands that personal problems may affect my performance.
	IMOC7	The division’s policies help meet employees’ individual needs.
	IMOC8	Management meets regularly to discuss issues related to employees’ challenges.
SA	SA1	My leader gives me authority over issues within my area.
	SA2	My leader listens to me.
	SA3	My leader encourages me to take the initiative.
	SA4	My leader is concerned that I shall work goal oriented.
	SA5	My leader instills motivation.
OA	OA1	(Hospital name) is attractive for me as a place of employment.
	OA2	I would recommend (hospital name) as an employer to my friends.
IIB	IIB1	Create new ideas to solve problems in my job.
	IIB2	Search out new working methods or techniques to complete my work.
	IIB3	Investigate and find ways to implement my ideas.
	IIB4	Promote my ideas so others might use them in their work.
	IIB5	Try out new ideas in my work.

*Note*: *SA* Support for Autonomy, *IIB* Individual Innovative Behavior, *OA* Organizational Attractiveness, *IMOC* Internal Market-Oriented Culture

### Data collection procedure

This study used a platform called Nettskjema (www.nettskjema.no) for data collection. The study participants were asked to consent to voluntary and anonymous participation. After several pretests and English–Norwegian back-translations had been completed, the final questionnaire was distributed via a link sent to the DOR, who forwarded it to division managers to distribute to their employees. With the Nettskjema platform, we were able to import the collected data to SmartPLS software for analysis. In the data collection process, the DOR was asked to forward several reminders to participate in the study to the division managers, who encouraged and reminded their employees.

### Data analysis

Partial least-squares structural equation modeling (PLS–SEM) was employed to test the conceptual models, using the software SmartPLS 3 [98]. The first step in evaluating PLS–SEM results involved examining the measurement model, which consisted only of reflective measures. The second step was to assess the structural model. Based on the PLS–SEM results, mediator effects were also estimated and analyzed. To test the mediator effect, the bootstrapping test of Zhao et al. [99] was used to assess whether the direct and indirect effects were statistically significant, and the combination of these two tests determined the degree of mediator effect. We followed the “rules of thumb” by Hair et al. [100, 101], to assess the quality of the measurement and structural model results.

### Ethical considerations

The study was submitted to and approved by the hospitals’ Data Protection Office (DPO) and The Norwegian Centre for Research Data (NSD), under project No. 239029, to comply with the research ethics guidelines set by the NSD. Participants were asked to consent to voluntary participation prior to the commencement of the survey. By taking advantage of the autonomous deletion of IP addresses that Nettskjema offers, the study and research project leader(s) were able to offer complete anonymity; the information was also validated and approved by the DOR and DPO.

## Results

### Measurement model

In an assessment of the reflective measurement model, we examined convergent validity, internal consistency reliability, and discriminant validity. Convergent validity is the extent to which item scores correlate positively with those of alternative items measuring the same construct and was evaluated from loadings of the items and average variance extracted (AVE). Internal consistency reliability was evaluated from the intercorrelations of the observed item scores within a construct and with composite reliability and Cronbach’s alpha. Discriminant validity is the extent to which a construct is distinct from other constructs and is assessed in this study from the heterotrait–monotrait (HTMT) ratio of correlation between constructs. The HTMT test reveals whether the HTMT value is significantly different from 1, or more precisely, whether the 95% confidence interval of the HTMT statistic did not include the value of 1. As can be seen in Table 4, all “rules of thumb” criteria by Hair et al. [102] have been met, providing confidence that this measurement model is both reliable and valid.

**Table 4:** Results of the measurement model for the IMOC, SA, OA, and IIB constructs

Latent variable	Item label	Convergent validity		Internal consistency reliability		Discriminant validity
		Indicator reliability	AVE^a^	Composite reliability	Cronbach’s alpha	HTMT criterion^a^
Rule of thumb		Loading > 0.7	> 0.5	0.7–0.95	0.7–0.95	HTMT interval does not include 1
IMOC	IMOC1	0.84	0.73	0.95	0.94	Yes
	IMOC2	0.76				
	IMOC3	0.89				
	IMOC4	0.86				
	IMOC5	0.90				
	IMOC6	0.84				
	IMOC7	0.82				
	IMOC8	0.90				
SA	SA1	0.84	0.80	0.95	0.93	Yes
	SA2	0.93				
	SA3	0.93				
	SA4	0.85				
	SA5	0.92				
OA	OA1	0.96	0.93	0.95	0.93	Yes
	OA2	0.96				
IIB	IIB1	0.86	0.77	0.94	0.92	Yes
	IIB2	0.88				
	IIB3	0.89				
	IIB4	0.88				
	IIB5	0.87				

^a^*AVE* Average variance extracted, *HTMT* Heterotrait–monotrait ratio of correlations, *SA* Support for Autonomy, *IIB* Individual Innovative Behavior, *OA* Organizational Attractiveness, *IMOC* Internal Market-Oriented Culture

### Structural model

**Fig. 2:**
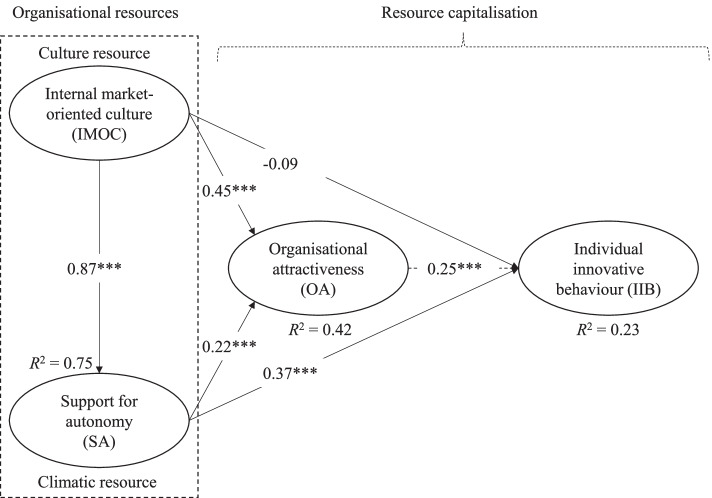
Results of the structural model to analyse the relationships between organisational resources and resource capitalisation. Standardized coefficients (*** < 0.01)

Before the structural model was assessed, multicollinearity between the latent constructs was determined from the variance inflation factor (VIF) values. VIF values above 5 indicate multicollinearity issues [103]. All VIF values were lower than 4, indicating no multicollinearity problems. The direct effects in the structural model are shown in Fig. 2. For the endogenous constructs, the model’s in-sample predictive power was examined using *R*^2^. Based on the rules of thumb, the *R*^2^ value was strong for SA (0.75), moderate for OA (0.42), and weak for IIB (0.23). Acceptable *R*^2^ values depend on the context, and in some cases an *R*^2^ of 0.10 is considered satisfactory [101, 103]. It is important to note that the results of the direct-path relationships are presented first, followed by the results of the proposed mediating relationships.

All the standardized direct-path coefficients were statistically significant at the 1% significance level, except the nonsignificant coefficient between IMOC and IIB. The direct-path coefficient between IMOC and SA was the highest (*β* = 0.87); the second highest was between IMOC and OA (*β* = 0.45) and the third highest between SA and IIB (*β* = 0.37). The direct relationship between SA and IIB was positive (*β* = 0.37), supporting H1. H2 and H4 were also supported, because the direct relationships between SA and OA and between IMOC and SA were significant and positive (*β* = 0.22 and *β* = 0.87, respectively). There was a nonsignificant direct relationship between IMOC and IIB (*β* = − 0.09), so H7 received no support. Finally, there was a positive direct relationship between IMOC and OA (*β* = 0.45), supporting H8.

We used the bootstrapping test of Zhao et al. [99] to test mediation. Briefly, this test uses bootstrapping to assess whether the direct and indirect effects are statistically significant, and the combination of these two tests determines whether there exist only direct effects—without mediation, no-effect nonmediation, complementary mediation, competitive mediation (direct and indirect effects are significant but in the opposite direction), or indirect-only mediation. The tests of the mediator effect show that OA complementarily mediates the relationship between SA and IIB, with a significant indirect effect of *β* = 0.06 (Table 5), supporting H3. SA complementarily mediates the relationship between IMOC and OA (the indirect effect of *β* = 0.19), supporting H5. The direct effects between IMOC and IIB were not statistically significant, implying that complementary mediation was not possible for H6 or H9. Our findings show that the statistically significant positive indirect effect of SA between IMOC and IIB was *β* = 0.32, indicating an indirect-only mediator effect, supporting H6. OA showed a significant positive indirect effect (*β* = 0.11) and an indirect-only mediator effect between IMOC and IIB, so H9 received support. A summary of the hypotheses leading this study and whether they were supported is shown in Table 6.

**Table 5:** Test of mediator effects of OA and SA

Hypothesis	Effect	Mediator	Direct effect^a^	Indirect effect^a^	Mediator effect^b^
H3	SA → IIB	OA	0.369^***^	0.055^***^	Complementary
H5	IMOC → OA	SA	0.446^***^	0.191^***^	Complementary
H6	IMOC → IIB	SA	−0.092	0.319^***^	Indirect-only
H9	IMOC → IIB	OA	−0.092	0.110^***^	Indirect-only

^a^ *** *p* < 0.01 is the significance level.

^b^ Mediation by bootstrap [99]

*SA* Support for Autonomy, *IIB* Individual Innovative Behavior, *OA* Organizational Attractiveness, *IMOC* Internal Market-Oriented Culture

**Table 6:** Results of hypotheses leading this study

Hypothesized relationships		Supported
H1	SA is positively related to employee IIB.	Yes
H2	SA is positively related to OA.	Yes
H3	OA mediates the relationship between SA and employee IIB.	Yes
H4	The IMOC is positively related to SA.	Yes
H5	SA mediates the relationship between an IMOC and OA.	Yes
H6	SA mediates the relationship between an IMOC and employee IIB.	Yes
H7	An IMOC is positively related to employee IIB.	No
H8	An IMOC is positively related to OA.	Yes
H9	OA mediates the relationship between an IMOC and employee IIB.	Yes

*Note*: *SA* Support for Autonomy, *IIB* Individual Innovative Behavior, *OA* Organizational Attractiveness, *IMOC* Internal Market-Oriented Culture

## Discussion

This study responds to the call for more research on intangible resources by focusing on organizational culture resources, IMOCs, and an organizational climate resource, SA. To the best of the authors’ knowledge, this is pioneering health services research that both includes and specifically examines two organizational resources (IMOC and SA) in a hospital setting. By examining the direct and indirect relations of IMOC and SA, this study both extends and deepens understanding and knowledge on the role of these intangible resources in healthcare services research. Specifically, this study answers the call of Carlucci and Schiuma [104] for further knowledge “about the role and the value of intangible resources in performance improvement” in research in healthcare organizations.

In line with the conceptual model of the study, as shown in Fig. 1, the aim of this study has been to examine the effect of organizational culture, IMOC, and leadership climate, SA, on hospital employees’ IIB. Organizational climate includes “rather superficial elements such as employees’ reactions, opinions and tendencies regarding changing or conflictual organization contexts” [104]. In this study, the climatic conditions, SA, concern leadership being supportive of employees. Specifically, SA focuses on the domain of work context and purposely on the conditions regarding employee work roles. SA refers to the interpersonal relationship between employees and their direct leader and whether this interpersonal work context is perceived or considered to be motivating, appealing, and encouraging [47]. As the findings from this study reveal, it is the climatic (organizational) resource provided by leaders’ SA that is capitalized on in different ways in hospital organizations.

This study found that SA has a direct impact on employee IIB (*β* = 0.37). The findings are in line with those in a recent study by Cho and Song [54] and Mutonyi [105] who examined the role of organizational culture on IIB. However, Cho and Song [54] focused on cooperative organizational culture and its role in Korean public employees’ IIB, whereas Mutonyi [105] focused on organizational culture type—clan or market—and its relation to the IIB of employees in higher education. As such, the current study offers new insights and knowledge into these relations in health services research.

Of all factors proposed to be directly related to IIB, the impact of SA was found to be the most influential. To the authors’ knowledge, this is one of the first studies in health services research to examine this relationship. These findings illustrate the importance of supportive leadership in a hospital organization, as it strongly triggers, stimulates, and promotes the capability of employees to act innovatively in their respective work roles. As such, the findings reveal a strong relationship between SA and IIB and underline the statement of Hocine and Zhang [77] that “people are most creative [an implicit part of IIB] when they feel motivated.” Both concepts connect with the idea of CoP communities for knowledge sharing and creation, a basis for innovation in organizations for designing a framework to systematically evaluate CoPs for their effectiveness in improving all practices and their capability to sustain improved practice initiatives [71, 72]. More precisely, CoPs are defined by three key interconnected features: mutual engagement (the amount and pattern of interactions in which individuals engage) by the members, the negotiation of a joint enterprise, and the development of a shared repertoire [106]. However, early indications show that in terms of IIB, CoPs alone or as part of larger interventions may have a role in improving healthcare performance [1, 9, 107].

Improving the performance of healthcare organizations requires a change of behavior among employees. This study shows that SA has a direct impact on employees’ cognition and OA, although SA has an impact on behavioral outcomes manifested in their IIB. Specifically, the study found that SA has a direct relationship with employees’ perceptions of OA (*β* = 0.22). OA in this study is considered to be a cognitive construct and refers to employee attitudes to the attractiveness of being employed in this hospital organization. In the article by Slåtten et al. [24], the authors state that “future research could include other factors that potentially promote OA” and specifically recommend examining the effect of leadership styles. In answer to this call, this study contributes new knowledge about the impact of SA on employee perceptions of OA in a hospital setting. Consequently, SA as an organizational climatic resource is an important driver of employee attitudes toward the OA of their employer. Thus, the relationship revealed in this study finds support in the work of Mesfin et al. [58], who state, “organizational climate [in this study manifested in SA] has a strong influence on employees.”

A substantial body of research has confirmed the importance of SA in promoting positive outcomes. For example, previous research has revealed that the effect of SA improves positive feelings toward a work activity [108, 109]. Furthermore, Gagne et al. [110] later found that autonomy support predicted trust in the organization and increased acceptance of organizational change. A supporter of autonomy would typically provide a good rationale for asking someone to engage in an activity and convey confidence in the person’s abilities [111]. This study dives deeper and contributes new knowledge and understanding on the key role of SA in health research. Specifically, this study found SA to be directly related to both employees’ IIB and OA. In addition, this study reveals that the attitude reflected in the concept of OA has a mediating role between SA and IIB. Specifically, based on the test undertaken in this study of the mediator suggested by Zhao et al. [99], it was found that OA functions as what Zhao et al. [99] label “complementary mediation.” Explicitly, this means that there are two routes of impact that do not substitute for each other but rather are simultaneously capable of having an impact on the level of employees’ IIB in hospital organizations. Specifically, one route goes directly from SA to IIB and the second works indirectly from SA via OA and eventually has an impact on employee IIB. The important role of OA in this relationship in hospital organizations is well formulated by Trybou et al. [25] who state that “hospital attractiveness is of major importance.” In a similar vein, Slåtten et al. [24] suggest that OA is “something that needs to be focused on, maintained, and cultivated if they [hospital managers] are serious about making their workplace highly attractive in a very competitive market.”

On broadening the current understanding of the organizational culture resource, the IMOC, the findings of this study reveal that it has a powerful impact on SA of leaders (*β* = 0.87). The IMOC explains 75% of the variance in SA. This is one of the first health services studies that examines the impact of an IMOC on SA in a hospital setting. The findings of this study highlights that IMOC “provides the rules for behavior” [4] for leaders providing SA. Furthermore, it shows how an organizational culture resource, an IMOC, can form and shape the organizational climatic resource, SA, in an organization. The findings of this study are in line with the meta-analysis by Slemp, Kern [112], who asserted the importance of understanding organizational culture and its effect on leadership autonomy support at work.

This study also revealed that the IMOC was related to the formation of employees’ perceptions of whether the hospital organization is a great place to work (referring to OA). The findings of this study are in line with those of Slåtten et al. [24] who also found a positive relationship between IMOC and OA in a study of hospital employees. However, this study extends previous research of the impact of IMOC by examining the different patterns of relationships associated with OA. Specifically, the direct impact of IMOC on OA was found to be twice that of SA on OA (respectively, *β* = 0.45 versus *β* = 0.22). Furthermore, in addition to the direct relationship, this study also found that the IMOC has an indirect impact via SA on OA. Specifically, based on the mediator test suggested of Zhao et al. [99], it was found that SA has the function of complementary mediation [99]. Complementary mediation implies that two pathways simultaneously lead to OA. In total, the direct or indirect impact of IMOC collectively explains 42% of the variance of OA. Consequently, this finding indicates that “organizational culture [in this study represented by IMOC] can be best described as a critical first step toward creating satisfactory work environments” [59].

Based on ideas and suggestions in previous research [24], it was anticipated that IMOC should have a direct impact on IIB. Surprisingly, the results identified a nonsignificant direct relationship between IMOC and IIB. On the other hand, the statistical analyses revealed a relatively complicated and multifaceted pattern that explains the links between the IMOC and IIB. Specifically, it was found that this relationship works through what is understood to be an “indirect-only” mediator effect [99]. Two indirect-only mediating effects of IMOC on IIB were identified, one that works through SA and another through OA.

### Practical implications

This study offers four practical implications for hospital managers.

First, as this study has shown, SA is an imperative organizational climatic resource. Consequently, SA may be labeled the first step by which hospital organizations can capitalize on both the relationship to employee IIB and perceived OA. This contribution to the SA literature has important implications for hospital managers. For example, to improve IIB, hospital management teams must continuously invest time and energy to develop and strengthen the SA of leaders. Team leaders have a significant impact on creating conditions that support team processes [113]. They must ensure that their staff members do not become too comfortable with formal procedures and practices to stimulate their employees’ habits and organizational routines, giving them sufficient autonomy for creating solutions to new daily problems. Such investments can be made in multiple ways: (i) through regular internal workshops in the respective hospitals, (ii) with a leadership training program in cooperation with external actors (e.g., universities), and (iii) with open feedback–reflection–action seminars where both leaders and representatives from employees participate, or (iv) by discussing the best SA practice in the organization. In addition, hospital managers could develop SA using confidential and standard employee surveys that are repeated regularly. An advantage of using standard surveys is that they enable tracking of potential changes and trends in employee experiences of SA from their leaders in the organization. Moreover, no matter how the investment in SA is done, the overriding goal should be to help leaders become more conscious of and progress in performing and delivering SA in a way that is beneficial for the hospital organization. Finally, developing CoPs to share and create knowledge and to stimulate mutual engagement between individuals entails that hospital managers must create key performance indicators for evaluating the dynamics to improve all practices and the capability to sustain these improvements [72]. As this study indicates, hospitals that absorb the cost of investing in SA will attain substantial returns because it capitalizes on both positive growth in employee perceptions of hospital OA while directly and indirectly (through OA) increasing employee IIB.

Second, as described above, SA is a significant organizational climatic resource for hospital managers to consider. However, as this study reveals, organizational culture is also a significant organizational resource. Some would even say that organizational culture fundamentally holds more importance than organizational climate. As Carlucci and Schiuma [104] noted, “climate can be understood as a surface manifestation of culture.” Similarly, Banaszak-Holl et al. [59] noted that organizational culture “pervades all aspects of organizational life.” Furthermore, organizational culture is said to be a “basic managerial tool for improving the work environment” [59] that is initiated and promoted by senior management. Organizational culture in this study has been represented by the concept of IMOC, which reflects one that is “purposely or intentionally directed toward employees in the organization” [10]. The results provide empirical support for the view that IMOCs are important for hospital organizations in several ways. For example, hospital managers are advised to offer opportunities for employees to discuss their needs and to align training to meet individual needs, which not only shows interest in training but also in the challenges faced by employees at work. In addition, for hospital managers to address such challenges, they are advised to familiarize themselves with their employees to improve their understanding of personal problems that may affect overall performance and clarify policies to meet their individual needs. Hospital managers can offer regular strategic meetings to provide a venue for discussions on issues related to employee challenges.

Third, hospital managers need to recognize that it is only when hospital employees have a positive view of the OA and SA in their organization that they are willing and motivated to devote the necessary time and energy to the extra-role efforts included in IIB. When leaders only intervene during times of turbulence, they will have only a narrow focus [114]. Therefore, gaining a broader view of employee understanding and leadership, especially in hospital teams or divisions, is about identifying and solving problems to ensure their effectiveness [115]. Therefore, hospital managers are encouraged to transfer some of their authority to their employees, especially in work tasks that fall into the employees’ area of expertise. Moreover, hospital managers can encourage employees to be proactive in taking initiative in implementing novel ideas at work. This also applies to instilling motivation while showing concern for the accomplishment of goals set by the hospital division or team. These practical implications will better prepare health organizations to improve OA to their employees.

Fourth, hospital managers should continually recognize that it is insufficient to focus solely on an IMOC in isolation. Hospital managers should consider an IMOC to be a basic managerial tool [59]. Findings from this study show that hospital managers should strive to understand IMOCs from a broader employee perspective. Specifically, they should continuously update their knowledge about how IMOCs can have a positive impact on the “inner life” of their employees in the hospital organization (referring to OA and SA). To innovate using the sharing and creation of knowledge found in CoPs, hospital managers must ensure that their staff members do not become too comfortable with the formal procedures, practices, and technical standards in place [116]. For example, hospital organizations are often found in complex environments [8] that hamper the creation of new ideas to solve problems at work, search out new techniques or working methods, promote ideas at work, or try them out. Consequently, a broader employee perspective on IMOCs while investing to uphold, cultivate, and develop an IMOC, will in tandem with SA lead to positive resource capitalization manifested in both improved employee perceptions of OA as well as their IIB.

In conclusion, this study contributes new knowledge regarding how organizational culture, IMOCs, and organizational climate, SA, function as two types of organizational resources, as well as their relation to OA and IIB. The study found SA to correlate with hospital employees’ OA and IIB, in addition to having an indirect effect. In addition, the study revealed IMOCs to correlate with SA and OA, with indirect effects on IIB. Thus, the findings of this study highlight the significance of investment in both IMOCs and SA by hospital managers. Based on these findings, hospital managers should make resource investments in IMOC and SA, as the payoff or resource capitalization will be manifested in both positive growth in employees’ perceptions of OA as well as employees’ level of innovative behavior. These are desirable outcomes for hospital managers and organizations that seek sustainable ways to use employees’ IIB at work.

### Limitations and future research

There are several opportunities for future research based on the limitations of this study. The following four areas of study are recommended.

First, this study is limited by its cross-sectional design. For instance, the empirical data in this study were collected at one point in time from a single health organization. Consequently, the results of this study should not be generalized to other health organizations. Regardless, the results can serve as a stepping-stone to future research including various health organizations, including testing causal and reversed casual relationships. This was done to minimize method bias. In addition, the limitations linked to online surveys are known to include self-selection and shared response bias, owing to the nature of self-report measures. Consequently, as suggested by Hair et al. [100], these limitations may reveal that future studies should employ a time lag in data gathering.

Second, this study is limited to the use of an IMOC to represent the organizational culture resource. However, future research should examine other types of potential cultural resources in hospital organizations. For example, one could include those culture types mentioned in the framework of Cameron and Quinn [117]. In their framework, the authors propose four types of organizational culture that could exist or dominate organizations: clan, adhocracy, market, and hierarchy. To the authors’ knowledge, few health services studies have examined their role. Therefore, it is highly recommended that future research focus on culture types in the Cameron and Quinn [117] framework and examine how they either individually or eventually collectively can achieve desirable objectives for hospital organizations. In addition to the new IMOCs, this study recommends that future research expand current knowledge and understanding of the complex nature of hospital environments, and the advantages of cultures that focus on employees.

Third, this study was also limited to SA to represent organizational climatic resources. As mentioned above, the domain of organizational climate as a concept requires the study of organizations based on the perspective of “how things are done here.” On this basis, there are numerous opportunities to include other climatic resources in hospital organizations in future research. Specifically, studying climatic conditions from a leadership perspective could include leadership styles such as empowering leadership, transformational leadership, ambidextrous leadership, transactional leadership, charismatic leadership, and so forth. Furthermore, from an employee perspective, future research could examine climatic aspects such as cooperative climates, communication climates, learning climates, supportive climates, trusting climates, humorous climates, and so forth. These aspects of organizational climate, like types of organizational culture, could be studied individually or in combination. Parallel to this study, it would also be possible for future research to examine how organizational cultures such as IMOCs thrive under CoPs to assess their impact on improving hospital employees’ OA, IIB, and overall healthcare practices [118].

Fourth, as noted throughout this paper, there is little research on the two types of resource capitalization included in this study (OA and IIB). Considering the importance of OA and IIB for hospital organizations, there is a need to include both of these factors in future health services research. However, in addition to including OA and IIB, other types of resource capitalization could be included. For example, considering the seemingly growing competition among hospitals (e.g., public versus private hospitals) in many countries, future research could include the concept of competitive power. The concept of competitive power focuses on “a company’s relative advantage in the marketplace in comparison to its most relevant competitors” [119]. Competitive power is reflected such as by being “the first to introduce new services into the market, having more satisfied customers and hard for competitors to imitate firms’ service offerings” [119]. Organizational culture is proposed as a potential source of competitive advantage [120]. Including the concept of competitive power in health services research will reveal what type of organizational culture and associated organizational climate best explain the competitiveness of hospitals. Consequently, focusing on these and several other options offers the potential for new knowledge and insights, both theoretical and practical.

## Supplementary Information

**Additional file 1: Appendix 1.** Questionnaire developed for this study.

## Data Availability

The datasets used and/or analyzed during the current study are available from the corresponding author on a reasonable request.

## Abbreviations

CoPs: Communities of practice
RBV: Resource-based view
SDT: Self-determination theory
LMX: Leader–member–exchange
SCT: Social cognitive theory
DOR: Director of research
IIB: Individual innovative behavior
SA: Support for autonomy
IMOC: Internal market-oriented culture
OA: Organizational attractiveness
PLS–SEM: Partial least-squares structural equation modeling
AVE: Average variance extracted
HTMT: Heterotrait–monotrait
VIF: Variance inflation factor
NSD: The Norwegian Centre for Research Data
HRM: Human resource management
DPO: Data Protection Office
